# Prevalence of and risk factors for severe cognitive and sleep symptoms in ME/CFS and MS

**DOI:** 10.1186/s12883-017-0896-0

**Published:** 2017-06-20

**Authors:** Vageesh Jain, Amit Arunkumar, Caroline Kingdon, Eliana Lacerda, Luis Nacul

**Affiliations:** 1 Guy’s Campus, King’s College London School of Medicine, London, SE1 1UL UK; 2 University of California, San Francisco, School of Medicine, San Francisco, CA 94143 USA; 3 Faculty of Infectious and Tropical Diseases, CURE-ME Team, London School of Hygiene & Tropical Medicine Disability and Eye Health Group, International Centre for Evidence in Disability (ICED, K/490, Keppel St, London, WC1E 7HT UK

**Keywords:** ME/CFS, MS, Risk factors, Cognitive symptoms, Sleep, Severity

## Abstract

**Background:**

There are considerable phenotypic and neuroimmune overlaps between myalgic encephalomyelitis/chronic fatigue syndrome (ME/CFS) and multiple sclerosis (MS). While the precise aetiologies of both MS and ME/CFS are unclear, evidence suggests that deterioration in cognitive function is widely prevalent in patients with either condition. Little is known about differing risk factors or exposures, which may lead to severe cognitive or sleep symptoms. This study aims to gauge the extent of cognitive and sleep symptoms in ME/CFS and MS patients participating in the UK ME/CFS Biobank and identify the characteristics of those experiencing severe symptoms.

**Methods:**

This was a cross-sectional study of 395 UK ME/CFS Biobank participants, recruited from primary care and the community, using similar standardised protocols, and matched by age, sex and geographical area. Data were collected from participants using a standardized written questionnaire at clinical visits. Cognitive symptoms included problems with short-term memory, attention, and executive function. Sleep symptoms included unrefreshing sleep and poor quality or inadequate duration of sleep. All participants reported symptoms based on an ordinal severity scale. Multivariable logistic regression was carried out in the ME/CFS group to investigate socio-demographic factors associated with severe symptoms.

**Results:**

All cognitive and sleep symptoms were more prevalent in the ME/CFS group, with ‘trouble concentrating’ (98.3%) the most commonly reported symptom. Severe symptoms were also more commonly reported in the ME/CFS group, with 55% reporting ‘severe, unrefreshing sleep’. Similarly, in the MS group, the most commonly reported severe symptoms were sleep-related. Logistic regression analysis revealed that ME/CFS patients aged over 50 years were more than three times as likely to experience severe symptoms than those younger than 30 (OR 3.23, *p* = 0.031). Current smoking was associated with severe symptoms, increasing the risk by approximately three times (OR 2.93, *p* = 0.003) and those with household incomes of more than £15,000 per year were less likely to experience severe symptoms compared to those earning less than this (OR 0.31, *p* = 0.017).

**Conclusions:**

Cognitive and sleep symptoms are more common in ME/CFS patients than in MS patients and healthy controls, providing further support for existing evidence of central nervous system abnormalities in ME/CFS. Our findings suggest that people with ME/CFS who are smokers, or have a low income, are more likely to report severe cognitive and sleep symptoms. Future research should aim to develop strategies to prevent the progression of severe cognitive and sleep symptoms through early interventions that prioritise patients identified as being at highest risk.

## Background

Myalgic encephalomyelitis/chronic fatigue syndrome (ME/CFS) and multiple sclerosis (MS) are chronic neurological disorders characterised by a number of symptoms including debilitating fatigue and cognitive decline [1, 2]. Some authors have proposed that ME/CFS and MS may share common aetiological and pathophysiological mechanisms [3–7]. While the precise aetiologies of both MS and ME/CFS are unclear, evidence suggests that changes in cognitive function are a core component of the conditions [8, 9]. Cognitive dysfunction has an impact on everyday life and reduces perceived quality of life [10] and includes problems with short-term memory, concentration, attention, and processing, which often coincide with sleep symptoms. Sleep disturbance is commonly reported, and may have an impact on non-cognitive symptoms such as fatigue, in both ME/CFS and MS [11, 12].

There are considerable phenotypic and neuroimmune overlaps between ME/CFS and MS [5, 13]. The pathophysiological process underlying changes in cognitive function in MS relates to the development of plaques affecting the myelin sheath and therefore axonal transmission [9]. The underlying process leading to cognitive changes in ME/CFS is not known, but inflammatory oxidative and nitrosative stress pathways may play a role [14].

A recent appraisal highlighted an increasing body of literature evidencing neurocognitive impairment in people with ME/CFS [1]. The authors acknowledged that despite the lack of evidence on objective changes in sleep architecture, unrefreshing sleep is omnipresent among those patients [1]. Thus, further insight into the severity of cognitive features in ME/CFS and MS will improve future diagnosis, case stratification, and management of these conditions.

Little is known about socio-demographic, behavioural and other exposures that may lead to severe cognitive and sleep symptoms, apart from potential links with life stress, which have been reported in distinct diseases [15, 16]. Consequently, Identifying risk factors for severe cognitive and sleep problems may aid in the development of methods to limit or prevent such debilitating symptoms.

This study aims to ascertain the prevalence of severe cognitive and sleep symptoms, and factors increasing the likelihood of reporting severe symptoms, in healthy controls and people with ME/CFS and MS participating in the UK ME/CFS Biobank.

## Methods

### Population and size

This was an analytical cross-sectional study with disease and control groups, who were recruited to the UK ME/CFS Biobank [17]. Of 395 participants, 237 had been diagnosed with ME/CFS, 47 with MS, and 111 were healthy controls. This sample size is adequate to identify prevalence differences of symptoms between people with ME/CFS or MS and healthy controls, with a power of 98% and of 91% respectively at a 0.05 significance level. This is based on a conservative prevalence of 3% for cognitive symptoms, among the general population [18]. The power calculation was performed with Epi Info 7™ [19]. The UK ME/CFS Biobank participants were recruited from those with a medically confirmed diagnosis of ME/CFS from National Health Services (NHS) in London, East Anglia and other parts of the UK, covered by the Clinical Research Network and Primary Care Network (CRN/PCN), local divisions and the ME/CFS pilot Disease Register (DR). A few severely affected participants were also recruited from support groups and patient associations. Healthy controls were frequency matched to cases by gender, geographical area of residence, and age within five years of cases. Healthy controls were excluded if they had previous or current chronic fatiguing disease, such as cardiomyopathy or cancer. Healthy controls were recruited through the PCN, invited by people with ME/CFS, or responded to adverts in the same areas of recruitment. MS patients were recruited from collaborating GP practices or other secondary care health services, particularly those in the CRN/PCN located in the same region as the ME/CFS cases. Standardised procedures, including inclusion and exclusion criteria and bespoke questionnaires applied to all consenting UK ME/CFS participants, are described in detail elsewhere [20].

### Inclusion and exclusion criteria

All participants, who were aged 18 to 60 years old, gave informed consent for study participation. ME/CFS cases were selected if they had a clinical diagnosis of ME/CFS according to the Centers for Disease Control and Prevention (CDC) [21] or Canadian Consensus Criteria (CCC) [8]. Disease status was confirmed by a clinical researcher, who reviewed laboratory test results to exclude alternative diagnoses. All MS cases had a diagnosis confirmed by a NHS neurologist, in compliance with the National Institute for Clinical Excellence (NICE) guidelines [22]. Information on specific type of MS was not routinely collected, but those on immunomodulatory drugs, were excluded due to the potential effects of these factors on disease processes and therefore reported symptoms. People with a diagnosis of MS were excluded if they had any other conditions apart from MS that could explain their fatigue including pregnancy or lactation.

### Data collection

Data were collected from all participants using self-completed questionnaires including validated instruments. Others were developed specifically for the recruitment of participants to the UK ME/CFS Biobank, to complement the clinical assessment, which included physical examination and blood collection. Data collection ran from March 2012 to December 2015 and consisted of demographic and socio-economic data and a range of exposure variables and symptoms. Socio-demographic details collected included age, gender, ethnicity, household income, and education. Lifestyle variables included smoking and drinking habits. Symptom assessments asked about cognitive, sleep, musculoskeletal, cardiovascular, autonomic, and systemic symptoms in the week prior to participant questionnaire. Co-morbidities and family health history were also recorded. The results presented in this paper focus on cognitive and sleep symptoms.

### Statistical analysis

Data were analysed using STATA 14 [23]. Cognitive symptoms analysed included problems with short-term memory, attention, and executive function; sleep disturbance was also recorded. Symptoms were analysed based on an ordinal severity scale (absent, mild, moderate, or severe) for three groups: ME/CFS, MS, and healthy controls. The prevalence of such symptoms in each group is displayed by severity. Chi-squared test was used in simple univariate analyses to compare non-continuous variables. Trends for statistical significance between symptoms across patient groups were tested with the *χ* ^2^ test for trend and the likelihood ratio test, considering “severity” as an ordered categorical variable [24]. A cut-off of 0.05 was used for the probability of alpha-1 error to indicate significance of an association.

Various factors were investigated for potential associations with severe cognitive and sleep symptoms in ME/CFS and MS. For the ME/CFS multivariable logistic regression model, all variables were analysed through univariate logistic regression and only included in the multivariable model if statistically significant or if considered to be clinically important. For MS, univariate logistic regression was used to investigate factors associated with severe cognitive and sleep symptoms because the small sample size precluded the use of a multivariable model.

Socio-demographic variables such as age and income were included in the final model because of a possible association between such factors and cognitive function [25, 26]. The low numbers of ethnic minority participants led to grouping of non-white ethnicities before comparing with white participants. Alcohol consumption six months before symptoms appeared in participants with ME/CFS or MS was classified as “no drinking” or “<14 units/week and ≥14 units/week”. This cut-off of 14 units/week was chosen because it is the current guideline for maximum weekly consumption recommended by the UK Department of Health [27]. Smoking status compared non-smokers to ex-smokers and current smokers. Other factors investigated in the initial univariate regression models included lifestyle factors such reported stress and activity levels as well as medical factors such as depression, anxiety, allergy, infection, asthma, and surgery in the six months before the questionnaire was completed. In the multivariable models, variables related to the time of recruitment, except for alcohol consumption and depression, which were also asked about six months previous to symptoms appearance. As duration of fatigue may have had an effect on the reported severity of symptoms, we also included this in the model, even if the difference was not statistically significant at the univariate analysis (Wilcoxon rank-sum test was used as this variable was not normally distributed). To limit the impact of missing data on the regression analysis, an assumption was made that any missing data for a specific variable corresponded to a “negative answer” or absence of the particular risk factor, providing a conservative estimate of the odds ratio. Missing data, although rare, was slightly higher in those with ME/CFS (1.42 per variable/100-persons) than those with MS (0.39 per variable/100 persons), and in healthy controls (0.25 per variable/100 persons).

The main dependent variable for the logistic regression analysis was a symptom severity score. This was calculated through an ordinal severity scale, with no points if the symptom was not reported, one point for a symptom reported as mild, two for moderate, and three for severe. An aggregate score (out of 27) for the severity of the nine individual cognitive and sleep symptoms was then calculated for each patient. Scores above the 75th centile for the ME/CFS group (21/27) and the MS group (14/27) were considered to be severe for each group respectively. Logistic regression was then used to investigate factors associated with severe cognitive and sleep symptoms in each of ME/CFS and MS groups by comparing those with scores equal to or above the 75th centile with those scores below it.

## Results

In the overall study population, 289 (73.2%) were female and 106 (26.8%) male. The median age was 44 years, with an interquartile range of 35 to 53 years. Three hundred and sixty-one participants (91.4%) identified themselves as white British, and 104 (28.3%) reported an income less than £15.000 a year at the time of data collection. Socio-demographic details of the study population are illustrated by group (Table 1).

**Table 1:** Characteristics of study population, by group

Variables	Categories	ME/CFS n (%)	MS n (%)	Healthy Control n (%)
Gender	Male	54 (22.8)	13 (27.7)	39 (35.1)
	Female	183 (77.2)	34 (72.3)	72 (64.9)
Age group	<30	40 (16.9)	0	18 (16.2)
	30–39	48 (20.3)	9 (19.1)	24 (21.6)
	40–49	65 (27.4)	15 (31.9)	30 (27.0)
	50+	84 (35.4)	23 (48.9)	39 (35.1)
Ethnicity	White British	220 (92.3)	45 (95.7)	96 (86.5)
	White Other	5 (2.11)	2 (4.26)	6 (5.41)
	Black African	1 (0.42)	0	1 (0.90)
	Asian	2 (0.84)	0	4 (3.60)
	Other	7 (2.95)	0	4 (3.60)
Income	<£15,000	71 (32.3)	10 (24.4)	23 (21.5)
	> = £15,000	149 (67.7)	31 (75.6)	84 (78.5)
Alcohol intake^a^	No Alcohol	87 (36.7)	9 (19.15)	25 (22.5)
	<14 units/week	118 (49.8)	27 (57.4)	69 (62.1)
	≥14 units/week	32 (13.5)	11 (23.4)	17 (15.3)
Smoking status	Never Smoked	150 (63,2)	19 (40.4)	78 (70.2)
	Ex-smoker	62 (26.1)	15 (31.9)	0
	Current Smoker	25 (10.5)	13 (27.7)	33 (29.8)
Depression^a^	No depression	143 (60.3)	36 (81.8)	111 (100)
	Depression	94 (39.7)	11 (23.4)	0

^a^Six months before symptoms for ME/CFS or MS (or at recruitment for healthy controls)

Table 2 displays the prevalence of cognitive and sleep symptoms over the previous week as reported by each group, by severity. All symptoms were more prevalent in the ME/CFS group, with ‘trouble concentrating’ the most commonly reported symptom in this group, at 98.3%. The remaining cognitive and sleep symptoms were common at over 80% for all except disorientation, which was reported by 56.5% of the ME/CFS group. Although not as frequent as in ME/CFS, cognitive and sleep symptoms were widely reported in MS patients, with the most commonly reported symptoms being unrefreshing sleep (78.7%), trouble concentrating (74.5%), problems with sleep quality or duration (72.3%), and short-term memory problems (72.3%). Healthy controls reported very few symptoms.

**Table 2:** Severity of cognitive and sleep symptoms over past week, by group

Symptom over past week	Severity	ME/CFS n/total = 237 (%)	MS n/total = 47 (%)	Healthy control n/total = 111 (%)	*P*-value*
Poor sleep quality/duration					<0.001
	Absent	26 (10.9)	14 (29.8)	70 (63.1)	
	Mild	31 (13.1)	15 (31.9)	26 (23.4)	
	Moderate	81 (34.2)	10 (21.3)	13 (11.7)	
	Severe	99 (41.8)	8 (17.0)	2 (1.8)	
Unrefreshing sleep					<0.001
	Absent	11 (4.6)	10 (21.3)	64 (57.7)	
	Mild	22 (9.3)	12 (25.5)	34 (30.6)	
	Moderate	73 (30.8)	12 (25.5)	11 (9.9)	
	Severe	131 (55.3)	13 (27.7)	2 (1.8)	
Difficulty retaining information					<0.001
	Absent	21 (8.9)	16 (34.0)	93 (83.8)	
	Mild	70 (29.5)	17 (36.2)	18 (16.2)	
	Moderate	83 (35.0)	11 (23.4)	0	
	Severe	63 (26.6)	3 (6.4)	0	
Difficulty understanding/thinking clearly					<0.001
	Absent	27 (11.4)	23 (48.9)	97 (87.4)	
	Mild	77 (32.5)	12 (25.5)	14 (12.6)	
	Moderate	76 (32.1)	9 (19.1)	0	
	Severe	57 (24.1)	3 (6.4)	0	
Slow thinking					<0.001
	Absent	34 (14.3)	19 (40.4)	97 (87.4)	
	Mild	77 (32.5)	14 (29.8)	14 (12.6)	
	Moderate	80 (33.8)	11 (23.4)	0	
	Severe	46 (19.4)	3 (6.4)	0	
Disorientation					<0.001
	Absent	107 (45.1)	31 (66.0)	107 (96.4)	
	Mild	51 (21.5)	9 (19.1)	3 (2.7)	
	Moderate	48 (20.3)	6 (12.8)	1 (0.9)	
	Severe	31 (13.1)	1 (2.1)	0	
Trouble concentrating					<0.001
	Absent	6 (2.5)	12 (25.5)	84 (75.7)	
	Mild	54 (22.8)	15 (31.9)	23 (20.7)	
	Moderate	102 (43.0)	18 (38.3)	3 (2.7)	
	Severe	75 (31.6)	2 (4.3)	1 (0.9)	
Short term memory problems					<0.001
	Absent	22 (9.3)	13 (27.7)	90 (81.1)	
	Mild	65 (27.4)	17 (36.2)	19 (17.1)	
	Moderate	91 (38.4)	15 (31.9)	2 (1.8)	
	Severe	59 (24.9)	2 (4.3)	0	
Brain fog/confusion					<0.001
	Absent	38 (16.0)	21 (44.7)	99 (89.2)	
	Mild	62 (26.2)	9 (19.1)	11 (9.9)	
	Moderate	77 (32.5)	15 (31.9)	1 (0.9)	
	Severe	60 (25.3)	2 (4.3)	0	

Missing data entered as ‘absence of symptoms’ for all groups

* χ^2^ test for trend

Severe symptoms were more commonly reported in the ME/CFS group, most notably unrefreshing sleep (55.3%) and poor quality or short duration of sleep (41.8%). Similarly, in the MS group the most commonly reported severe symptom was unrefreshing sleep (27.7%). The number of MS patients reporting severe non-sleep related symptoms was low ranging from 2.1 to 6.4%, compared with the ME/CFS group where it ranged from 13.1 to 31.6%. We have compared the duration of fatigue between people with ME/CFS (median = 11.1 years; Inter Quartile Range (IQR) 5.9 to 17.6), and people with MS (median = 10.7 years; IQR = 6.4 to 19.3), using a non-parametric test and the difference was not significant (*p* = 0.688).

## Logistic regression analysis

Table 3 displays the results from the multivariable logistic regression model of the factors affecting severity of cognitive and sleep symptoms in ME/CFS patients. No clear trend could be observed between increasing age and symptom severity, although patients aged over 50 were more than three times more likely to experience severe cognitive and sleep symptoms compared with those less than 30 years old (OR 3.23, *p* = 0.031). Current smoking increased the risk of severe cognitive and sleep symptoms by approximately three times (OR 2.93, *p* = 0.003) and ex-smokers were more than twice as likely to experience symptoms compared with non-smokers (OR 2.17, *p* = 0.034). People with household incomes of £15,000 or more per year were approximately 70% less likely to report severe symptoms compared to those earning less than this (OR 0.31, *p* = 0.017). There were no statistically significant associations between gender, ethnicity or depression and severe cognitive and sleep symptoms in ME/CFS.

**Table 3:** Multivariable Analysis: Factors related to prevalence of severe cognitive and sleep symptoms in ME/CFS

Variable	Odds ratio	95% Confidence interval	*p* value
Age < 30	1.00	-	0.204
Age 30–39	2.30	0.69–7.66	
Age 40–49	2.34	0.73–7.56	
Age 50+	3.23	1.04–10.04	
Male	1.00	-	0.138
Female	1.88	0.81–4.30	
Non-white ethnicity	1.00	-	0.521
White ethnicity	0.65	0.18–2.39	
Income < £15,000	1.00	-	0.017
Income > = £15,000	0.31	0.12–0.81	
No alcohol (six months before)	1.00	-	0.942
Alcohol <14 units/week (six months before disease onset)	0.96	0.48–1.94	
Alcohol ≥14 units/week (six months before disease onset)	1.15	0.40–3.30	
No smoking	1.00	-	0.026
Ex-smoker	2.17	1.06–4.42	
Current smoker	2.93	1.11–7.70	
No depression before symptoms	1.00	-	0.169
Depression before symptoms	1.58	0.82–3.03	
Duration of fatigue (years)	0.99	0.94–1.05	0.804

Table 4 shows logistic regression analysis results for the factors affecting severity of cognitive and sleep symptoms in patients with MS. The only variable significantly associated with severe symptoms in MS was previous depression. MS patients who experienced depression prior to symptoms were over seven times more likely to report severe symptoms (*p* = 0.009). Socio-demographic variables including age, gender, ethnicity, income, smoking, and alcohol consumption were not associated with severe cognitive and sleep symptoms in MS.

**Table 4:** Univariate analysis: factors related to the prevalence of severe cognitive and sleep symptoms in MS

Variable	Odds Ratio	95% confidence interval	*p* value
Age < 30	1.00	-	0.731
Age 30–39	0.94	0.18–4.79	
Age 40+	0.47	0.10–2.16	
Male	1.00	-	0.424
Female	0.58	0.15–2.23	
Income < £15,000	1.00	-	0.756
Income > = £15,000	0.67	0.05–8.64	
No alcohol (six months before)	1.00	-	0.21
Alcohol <14 units/week (six months before disease onset)	0.53	0.11–2.49	
Alcohol ≥14 units/week (six months before disease onset)	0.28	0.04–2.09	
No smoking	1.00	-	0.93
Ex-smoker	0.79	0.18–3.53	
Current smoker	0.96	0.21–4.42	
No depression before symptoms	1.00	-	0.009
Depression before symptoms	7.25	1.65–31.8	

## Discussion

### Key findings

Cognitive and sleep symptoms were more common in ME/CFS patients than in MS patients and healthy controls. The number of participants reporting severe symptoms was also highest in the ME/CFS group. The most commonly reported severe symptom in both groups was unrefreshing sleep: 55.3% in the ME/CFS group, compared with 27.7% in the MS group. The number of MS patients reporting severe non-sleep related symptoms was relatively low (ranging from 2.1 to 6.4%), compared with the ME/CFS group (ranging from 13.1 to 31.6%).

In the ME/CFS patients, multivariable logistic regression revealed that the severe cognitive and sleep symptoms were associated and increased with previous and current smoking as well as with low income (<£15,000). Although there was no consistently significant trend with age, those over 50 years were more likely to experience severe symptoms compared with those less than 30 years old. In the MS group, univariate logistic regression demonstrated that previous depression was associated with a higher likelihood of patients reporting severe cognitive and sleep symptoms.

### Prevalence of severe cognitive and sleep symptoms

The Canadian Consensus document [8] states that cognitive symptoms including confusion, slowed processing of information, difficulty concentrating, poor short-term memory, and sleep problems are common in ME/CFS. Fukuda et al. [28] propose that apart from fatigue and musculoskeletal pain, the most prominent features of ME/CFS are impairments in concentration, short-term memory, and sleep disturbances. Recently, the Institute of Medicine reviewed the evidence on the major symptoms of ME/CFS and reiterated that cognitive symptoms, sleep related symptoms and primary sleep disorders are common features [1]. Our study provides an empirical estimation of the proportion of ME/CFS patients affected by cognitive symptoms, with all symptoms except disorientation being reported in more than 80% of ME/CFS patients. We also found that the classic ME/CFS symptoms of sleep disturbance and trouble concentrating were most commonly reported as severe compared to other symptoms.

Unrefreshing sleep, or sleep of poor quality or short duration were the most commonly reported severe symptoms in both the ME/CFS and MS groups. Krupp et al. evaluated the sleep characteristics in 72 patients with ME/CFS compared with 57 MS patients [11] and found that the ME/CFS patients experienced significantly more sleep disturbance compared with MS patients. Among the ME/CFS patients, 37% reported light sleeping, 15% reported inadequate sleep, and 27% reported early waking, compared with 10, 5, and 0% respectively for the MS group. The findings of our study are consistent with rates were higher for ME/CFS participants, although sleep disturbances were common in both groups.

Guidelines for ME/CFS participant (Fukuda criteria) study inclusion advise excluding those with primary sleep disorders. Unrefreshing sleep is the only ME/CFS criteria relating to sleep in the Fukuda guidelines [28]. However, other authors argue that primary sleep disorders should be treated as co-morbidities, and their presence should not preclude a diagnosis of ME/CFS [1]. In people with ME/CFS, no consistent abnormal sleep pattern has been found on polysomnography [29] adding to the difficulty in treating sleep disturbances. Nevertheless, the emergence of new, more sensitive techniques suggest that alterations in sleep stage transitions and other physiological mechanisms, such as heart rate variability and cortisol profiles, may be evident in ME/CFS. A 2013 cross-sectional study of 343 ME/CFS patients [29] described four sleep-specific phenotypes, with objective differences among them in sleep onset latencies as well as rapid eye movement (REM) stages and latencies. The high prevalence of severe sleep symptoms reported in our study indicates that data characterising distinct sleep patterns could prove vital in identifying targeted methods for treatment in ME/CFS patients.

### Risk factors for severe cognitive and sleep symptoms

Our study is unique in exploring risk factors for cognitive and sleep symptoms and their severity in ME/CFS. A 2008 systematic review of eleven case-control, cohort, and cross-sectional studies assessed various potential factors correlated with the development of ME/CFS, but causal evidence is lacking [30]. Factors found to be significantly associated with the development of ME/CFS, which our study also investigated, were older age [31, 32], being female [32, 33], the presence of an anxiety disorder [31], mood disorder [34] or stress [35], and a history of allergies or asthma [36]. Based on our findings, the majority of these factors do not appear to translate from risk of development of ME/CFS to risk of severe cognitive and sleep symptoms in ME/CFS. That said, ME/CFS patients over the age of 50 were more than three times more likely to experience severe cognitive and sleep symptoms, compared to those under age 30. The reason for older age causing more severe symptoms are complex, but may be at least partially due to age-related decreases in brain dopamine activity contributing to impaired performance on tasks that involve frontal brain regions [37]. The pathophysiological processes occurring in ME/CFS may exaggerate normal age-related processes. For instance, Xenon-computed tomography blood flow studies show significantly lower cortical/cerebellar regional cerebral blood flow (rCBF) in ME/CFS [38].

A cross-sectional population-based US study (*n* = 5623) found that income was not related to the prevalence of ME/CFS in the general population [39]. However, our analysis found that ME/CFS participants with household incomes of under £15,000 per year were at an increased risk of severe cognitive and sleep symptoms. Although there scant literature exploring the effect of income on severity of symptoms in ME/CFS, a 1995 cohort study [40] of 49 patients found that environmental and psychosocial stressors exacerbated self-reported ME/CFS symptoms following a natural disaster. It is plausible that such stressors may play a similar role in the daily lives of individuals with lower incomes. In addition, existing literature describes an association between relative poverty and sleep symptoms in healthy individuals [41]. However, more evidence is required to explore the possible association between low income and severity of cognitive symptoms in ME/CFS, and the precise mechanism of this relationship.

Smoking was also found to be associated with severe cognitive and sleep symptoms. Previous evidence from a large multicentre cohort study (EURODEM), with 9209 non-demented patients selected for follow-up, found that smoking leads to cognitive decline [42]. The mini-mental state exam (MMSE) of those who never smoked declined by 0.03 points/year. The decline in score for previous smokers was 0.03 points greater than this, and for current smokers was 0.13 points greater than never smokers (*p* < 0.001). The results of our study concur with such evidence, finding a relationship between smoking habits and severe cognitive and sleep symptoms in ME/CFS. This is of clinical importance as it suggests that stopping or preventing smoking may help reduce the risk of developing more severe symptoms in such patients.

### Strengths and limitations

This study included a relatively large sample size of ME/CFS participants (*n* = 237), a healthy control group, and participants with another neurological disease for comparison. This enabled a reliable statistical analysis of predictive factors for people with severe symptoms of ME/CFS. The study population included participants from multiple sites across the UK and with varying degrees of severity, so although our results may not be generalizable to the entire UK population, they are reflective of a wide range of participants rather than a particular subset. All data were collected in a standardized form and all participants had contact with the clinical team for assessment, optimizing data reliability. Finally, data were gathered on a broad range of socio-demographic variables, exposures, and symptoms. For this reason, we have been able to explore the prevalence of cognitive and sleep symptoms between different disease groups and investigate numerous predictive factors.

The main limitation of the study lies in the relatively low number of participants with MS (*n* = 47), restricting our ability to conduct multivariable analysis in this group. Nonetheless, univariate regression analysis was used to explore potentially associated factors of clinical importance, but these will require validation in studies with larger sample sizes. In addition, information on specific type of MS was not routinely collected, and therefore we were not able to ascertain differences between distinct MS subtypes. In the overall study population, most of the participants recruited identified themselves as being of white British ethnicity (*n* = 391), restricting the generalizability of results to the wider multi-ethnic population. Although our analysis included severely-affected participants there may have been some people with ME/CFS who were so ill, for example on nasogastric feeding, that they could not participate in the study. Additional limitations inherent to the study design and data collection methods are recognized and have to be taken into account when considering our findings, such as the possibility of residual confounding. The cross-sectional nature of the data, even with a control group, can be indicative of associations, but not causality. Additionally, recall bias is also an issue to be considered in self-reported data collection. However, the consistency of responses across the three groups indicates that this bias is unlikely to have been significant.

### Implications for clinical practice and future research

In MS patients, a history of depression was found to be associated with severe cognitive and sleep symptoms. Depression is common in MS [43] and is thought to interfere with cognitive function. Arnett et al. [44] conducted a critical review of the existing literature on depression in MS, to find that a positive link has been consistently noted between depression and cognitive functioning. Depression affects many aspects of cognitive functioning in MS, including working memory, processing speed, and executive functioning [45–47]. Our findings therefore support existing evidence, and suggest that efforts aiming to treat or prevent depression could lead to improvements in the reported severity of cognitive symptoms in patients with MS.

The high prevalence of cognitive and sleep symptoms in ME/CFS highlights the need for improved strategies to combat their effects. Unlike MS, the severity of these symptoms seems to be unrelated to depression, and might reflect more generalised and subtle abnormalities in the CNS. Unrefreshing sleep was the most commonly reported severe symptom. Research has demonstrated that sleep physiology is altered in ME/CFS patients [48], but management of the condition, and available pharmacological therapy, still fails to address this critical issue. Future research must explore both pharmacological and non-pharmacological methods aimed at improving quality and duration of sleep in ME/CFS.

In clinical practice, patients may be advised of the various factors that can lead to severe cognitive and sleep symptoms. To recommend smoking cessation is a general public health measure that can be reinforced, and prevention of smoking in those at potential higher risk of ME/CFS, such as those with prolonged fatigue following an infectious disease, is important to reduce risk of severe cognitive and sleep symptoms. Public health and medical interventions to improve symptoms must recognise that those with a low-income (≤£15,000) seem to be at a higher risk of developing severe symptoms.

Despite the relatively high prevalence of cognitive impairment in MS, cognitive function is not assessed routinely in clinical practice or in clinical trials [49]. Our findings suggest that sleep problems, rather than other cognitive problems, are severe in a large minority of MS patients. The perception that cognitive assessments are costly, time-consuming and difficult to interpret has contributed to the failure to incorporate cognitive testing into standard clinical evaluation of patients with MS. Detailed studies of cognitive impairment in MS are rare, and guidelines for the assessment of cognitive or sleep function in MS are lacking. Future research must address these gaps in the current knowledge base. Researchers may find it beneficial to incorporate cognitive and sleep function measures into clinical assessment of MS and ME/CFS, and encourage the development of novel therapies targeting such symptoms.

## Conclusions

Cognitive and sleep symptoms were more common in people with ME/CFS in this study than in both healthy controls and people with MS. This provides further support for existing evidence of central nervous system abnormalities in ME/CFS and highlights the need for improved strategies to address the effects of such symptoms. Unrefreshing sleep was the most commonly reported severe symptom in both people with ME/CFS and people with MS, although it was more prevalent in those with ME/CFS. Our findings suggest that people with ME/CFS who are smokers, or have a low income, are more likely to report severe cognitive and sleep symptoms. Future research should aim to develop strategies to prevent the progression of severe cognitive and sleep symptoms through early interventions that prioritise patients identified as being at highest risk.

## Abbreviations

CCC: Canadian Consensus Criteria
CDC: Centers for Disease Control and Prevention
CRN/PCN: Clinical Research Network and Primary Care Network
DR: Disease register
IQR: Interquartile range
LSHTM: London School of Hygiene & Tropical Medicine
ME/CFS: Myalgic encephalomyelitis/chronic fatigue syndrome
MMSE: Mini-mental state exam
MS: Multiple sclerosis
NHS: National Health Service
NICE: National Institute for Clinical Excellence
NRES: National Research Ethics Service
OR: Odds ratio
rCBF: Regional cerebral blood flow
REM: Rapid eye movement
